# Complications of traditional bone setters (TBS) treatment of musculoskeletal injuries: experience in a private setting in Warri, South-South Nigeria

**DOI:** 10.11604/pamj.2018.30.189.15730

**Published:** 2018-07-02

**Authors:** David Odoyoh Odatuwa-Omagbemi, Thomas Odafe Adiki, Cornelius Itodo Elachi, Anirejuoritse Bafor

**Affiliations:** 1 Department of Surgery, Delta State University, Abraka, Nigeria; 2 Department of Orthopaedic Surgery, Central Hospital, Warri, Nigeria; 3 Department of Surgery, Benue State University, Makurdi, Nigeria; 4 Department of Orthopaedics and Traumatology, University of Benin, Benin City, Nigeria

**Keywords:** Traditional bone setters, musculoskeletal injuries, complications, private health facility, orthopaedic practice

## Abstract

**Introduction:**

Complications arising from the practice of traditional bone setting is a major contributor to the challenges the orthodox orthopaedic practitioner in Nigeria faces. We share our experience at a multi-specialist private health facility in Warri, South-south, Nigeria.

**Methods:**

Case notes of patients with musculoskeletal injuries who had prior treatment by traditional bone setters with resulting complications before presenting at our health facility for treatment were reviewed and relevant information extracted and entered in an already prepared proforma. Data were analysed using SPSS version 17 and results presented in form of means, percentages, ratios and tables.

**Results:**

43 cases were reviewed in a period of 8 years. There were 21 males and 22 females. The average age of patients was 44.8 ± 20.3 years. The most frequent age group affected was that of 40-49 years. 45.8% of the initial injuries were due to road traffic accidents while 39.5% resulted from falls. Femoral fractures and humeral fractures formed 20.4% and 14.8% of cases respectively. 40.8% of traditional bone setters complications observed were non-union of fractures of various bones followed by mal-union in 24.5% of cases.

**Conclusion:**

The observed complications of traditional bone setters practice in this study were similar to those previously reported in the literature. These complications constitute a significant challenge to the orthopaedic practitioner in Africa with associated negative socioeconomic impact on our society. Government and other relevant stakeholders need to unite and take decisive actions to mitigate this problem.

## Introduction

In many parts of the developing world, large proportion of fractures continue to be treated by traditional bone setters (TBS) who are readily available and often have a good local reputation [1]. In Nigeria, the practice of traditional bone setting is extensive and enjoys enormous patronage by the populace [2]. Up to 85% of patients with fractures are said to first present to traditional bone setters before presenting to orthodox hospitals [3]. This popularity is due to its long period of existence prior to the coming of orthodox practice, the ubiquity of the practitioners, affordability and acceptance of variable modes of payment (both in cash and kind) for services rendered in addition to wide spread belief in its effectiveness [2,4-7]. The practice like many other traditional practices, is passed on from generation to generation along family lines in form of apprenticeship with occasional admission of persons outside the family line to learn the trade also as apprentices. There is usually no formal training curriculum, no basic qualification and the level of competence varies widely which accounts for most of the problems encountered with their practice. In addition, there is no legislative or government control of TBS practice in Nigeria today [3,7-10]. Musculoskeletal injuries including fractures, sprains, dislocations etc, are manipulated (with skin scarification in some cases) and concoctions applied after which splinters are applied and the limb bandaged. In addition some traditional bone setters perform rituals with incantations to invoke divine assistance in the healing process. A few practitioners even go further to offer some form of orthodox care by administering analgesics, antibiotics and wound care in cases of open fractures with the assistance of some medical auxillaries [3,7,11-13]. In spite of this popularity, the practice of traditional bone setting has been associated with unacceptable outcomes in many cases. Extreme cases include limb gangrene and death largely from sepsis/septicaemia, tetanus and anaemia. Other reported complications include: mal-union and non-union of fractures, compartment syndrome, Volkmann's ischaemic contractures, conversion of closed to open fractures, osteomyelitis and soft tissue infections, chronic unreduced joint dislocations, joint stiffness and ankylosis, septic arthritis, pressures sores and blisters, iathrogenic fractures and other injuries [2-4,7,8,10,12,14-16]. Many of the studies and reports on complications of traditional bone setters (TBS) in the past have been from experiences from patients seen in government owned health facilities and occasionally mission hospitals. We present our experience on the pattern of complications of treatment of musculoskeletal injuries by traditional bone setters (TBS) as observed at a multi-specialist private health facility in Warri, South-south Nigeria.

## Methods

This is a retrospective study carried out in a multi-specialist private hospital in Warri, South-south, Nigeria. It involved a review of case notes of patients with musculoskeletal injuries who presented to the hospital with complications arising from prior treatment by traditional bonesetters over a period of 8 years (January 2009 to December, 2016). Information on patients' bio-data, type and aetiology of initial injuries, treatment offered by the bone setters, complications at presentation and treatment offered by the hospital were entered into an already prepared proforma designed for that purpose. Case notes with inadequate information were left out of the study. Data were analysed with SPSS version 17 soft ware and presented in form of means, ratios, percentages and tables.

## Results

**Figure 1:**
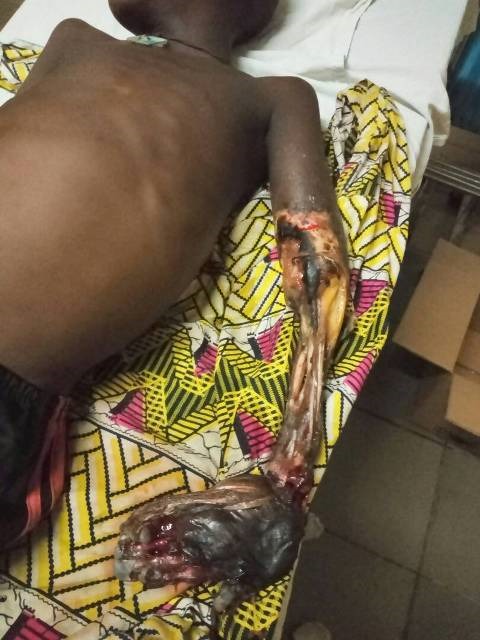
Left upper limb gangrene in a 7 year old boy from tbs treatment of an elbow injury

A total of 4,702 Othopaedics and trauma cases were seen in the hospital in the 8 years period under review out of which 43 patients (0.91%) presented with complications of traditional bone setters' prior treatment of their injuries. There were 21 males and 22 females (male : female ratio = 1 : 1.0^5^). The average age of patients was 44.8 ± 20.3. Age of patients ranged from 2-81 years. The age group that presented most frequently was 40-49 years (23.3%) followed by 30-39 years (20.9%) Table 1. Traders and business persons were most frequent affected making up 27.9% of the patients followed by those schooling at various levels (Table 1). In terms of aetiology of initial injuries, 24 patients (55.8%) had injuries due to road traffic accidents followed by injuries from falls in 17 patients (39.5%). One of the patient (2.3%) sustained injury from assault and another one (2.3%) from to sporting activities. Femoral fractures made up 20.4% of the initial injuries treated by traditional bone setters with resulting complications in this study Table 2. 40.8% of patients with TBS complications presented with non-union of fractures of various bones followed by mal-united fractures which formed 24.5% of cases. There were a total of 49 complications in 43 patients Table 3. Figure 1 is a photogragh of one of the children that had left upper limb gangrene from TBS treatment of an elbow injury who was treated with above elbow amputation. The time between injury and presentation to us ranged between 2 days in a two years old patient who had upper limb gangrene and 31 years in a 37 years old clergy man who had brachial plexus injury in childhood. Treatments offered to patients included: open reduction and internal fixation with or without bone grafting for cases of non-union and mal-union (after initial osteoclasis) of fracrures. Open reductions were done for chronic unreduced joint dislocations, while the two children with upper limb gangrene had amputations. Patients with stiff elbows and myositis ossificans had oral Indomethacin and physiotherapy. Antibiotics and wound care were given to the patient with infection and pressure sores and fasciotomy was done for compartment syndrome. Masterly inactivity was adopted for the patient with long standing brachial plexus injury after counselling on prognosis.

**Table 1:** Socio-demographic characteristics of patients

**Sex distribution of patients**		
sex	**frequency**	**percentage**
male	21	48.8
female	22	51.2
total	43	100
**Age distribution of patients in years**		
**Age group years**	**frequency**	**percentage**
0 - 9	3	7.0
10 - 19	2	4.7
20 - 29	3	7.0
30 - 39	9	20.9
40 - 49	10	23.3
50 - 59	5	11.6
60 - 69	5	11.6
70 - 79	5	11.6
80 - 89	1	2.3
**total**	43	100
**Occupational distribution of patients**		
occupation	**frequency (no)**	**percentage**
traders /business persons	12	27.9
schooling	6	14.0
retirees	5	11.6
unemployed	5	11.6
artisans / unskillled labour	4	9.3
clergy	3	7.0
civil servants	3	7.0
others	5	11.6
**TOTAL**	43	100

**Table 2:** Injuries treated by traditional bone setters (tbs) with resulting complications

Injuries	Frequency (no)	Percentage
Femoral fractures	11	20.4
Humeral fractures	8	14.8
Radial fractures	8	14.8
Tibial fractures	7	13.0
Ulnar fractures	7	13.0
Fibular fractures	4	7.4
Elbow disloations	4	7.4
Ankle fractures	2	3.7
Shoulder dislocation	1	1.9
Elbow soft tissue injury	1	1.9
Brachial plexus injury	1	1.9
Total	54	~100

**Table 3:** Types of TBS complications

TBS complications	Frequencies (n)	Percetages
Non union	20	40.8
Mal-union	12	24.5
Joint stiffness / myositis osificans	6	12.2
Chronic unreduced joint dislocations	5	10.4
Limb gangrene	2	4.1
Unreduced distal radial physeal injury	1	2.0
Neglected brachial plexus injury	1	2.0
Compartment syndrome	1	2
Pressure sores, blisters and local sepsis	1	2.0
Total	49	100

## Discussion

Complications arising from the practice of traditional bone setting (TBS) significantly contribute to the challenges facing the Orthopaedic practitioner. [16]. These complications range from less severe ones like minor limb length discrepancies from mal-union of fractures with minimal effect on function, to major ones like limb gangrene and death. Our findings in this study are similar to those reported in the literature by previoius authors [2,7,8,14,15,17,18]. 43 patients who took TBS treatment after injury before coming to us presented with 49 complications of varying degrees of severity. The most serious complication in this series was limb gangrene which occurred in two children who were offered above elbow and below elbow amputations respectively. In this study, there were 21 males and 22 females giving a male : female ratio of 1:1.05. The preponderance of female in this study sharply contrasts findings in several other previous studies on TBS complications where male folks predominate [5,10,15,17-19,20]. More studies need to be done to corroborate this observation and explain possible reasons for it since the preponderance of males in previous studies had always been explained by the undisputed fact that males by their nature and activities are usually more prone to injuries. The average age of patients in this study was 44.8+20.3 years (ranged from 2-81 years). This mean age is much higher than that observed in some previous studies [17,19,20]. Average ages of 36.8 years, 32.3 years and 28 years have been reported respectively by Onyemaechi et al [20], Kuubiere et al [17] and El Hag et al [19] respectively. Similarly the age group most frequently encountered in this study is 40-49 years which is also much higher than those observed in other studies [15,17,19,20]. Older patients probably are more able to afford treatment in a private setting and would prefer the one on one attention obtainable in such a settings. The frequencies of various TBS complications of treatment of musculoskeletal injuries appear to vary from study to study. However, malunion and non-union of fractures tend to feature significantly as being the most frequently observed complication in many previous studies including the present one. Kuubiere et al [17] and El Hag at al [19] reported mal-union as the most frequently observed complication in their series followed by non-union of fractures. Joint stiffness and infections both followed by mal-union of fractures were observed as the most frequently encountered TBS complications by Onyemaechi et al [20] and Nwandiaro et al [15] respectively. In this series fracture non-union was the most frequent complication followed by mal-union. A possible partial explanation for the observed variations may be the level of urbanisation in the environment of study, the nature and aetiology of the initial injuries, the competence and manner of practice of the traditional bone setters in a particular region and other socio-demographic factors which may need further investigations. Our study shows the femur as the most frequently fractured bone treated by TBS with resulting complications followed by the humerus and the forearm bones. This is similar to what has been reported by Kuubiere et al [17] from Ghana. In contrast, Nwandiaro et al [15] from North-Central Nigeria reported equal involvement of the femur, tibia/fibula, humerus and forearm bones while Onyemaechi et al [20] from the same region observed tibia as the commonest bone involved in TBS complications followed by the femur. Socio-demographic differences and other local factors may account for some of these variations. With regards to the aetiology of initial injuries treated by TBS with resulting complications in this study, more than 55% was due to road traffic accidents followed by falls. This is similar to findings by Onyemaechi et al [20] who reported that 69.4% of injuries in their study were due to road traffic accidents followed by falls in 27.8% of cases. On a general note, this finding is not surprising as road traffic accident has been reported in previous studies as the commonest cause of musculoskeletal trauma in our environment [21-23].

## Conclusion

From the above discussion, it is clear that the practice of traditional bone setting (TBS) is a major source of Orthopaedic complications with resulting morbidities and sometimes mortalities in our environment. The experiences of orthodox Orthopaedic practitioners in public hospitals are not quite different from what occurs in private health facilities as the complications are similar with only some variations in diagnostic frequencies and socio-demographic characteristics of patients. Trauma and its attendant complications generally affect more of the productive age group in the society as is also obvious in this study. The activities of TBS in Nigeria further compound this, making the complications worse and leading to prolonged periods of morbidity and impaired functions in those affected. The negative socioeconomic impact of TBS practice on developing countries like ours cannot thus be under estimated. It is as such imperative that all stakeholders including government and its agencies, the legislature, Orthopaedic practitioners, non-governmental organisations and advocacy groups should come together and find a lasting solution to this avoidable menace. The need for public enlightenment, TBS education and certification, legislative control of TBS practice and well spelt out penalties for mal-practice cannot be over emphasized.

### What is known about this topic

- Complications arising from TBS treatment of musculoskeletal injuries constutes a serious challenge to othopaedic practice in our environment;
- Major TBS complications incude: malunion and non-union of fracrures, chronic osteomyelities, limb gangrene, septicaemia and death;
- Most previous reports show male preponderance among the patients.

### What this study adds

- Experience on TBS complications from treatment of musculoskeletal injuries in a private sector health facility is not quite different from that in the government owned public health facilities;
- Major complications observed in this study include: Non-union of fractures (most commonly seen in this study), mal-unions, chronic neglected joint dislocations etc-the most severe type seen in this study being upper limb gangrene in two children;
- Female predominance in the patients presenting have been noted in this study.

## Competing interests

The authors declare no competing interests.
